# The Use and Preference of Functional Appliances among a Sample of Iraqi Orthodontists: A Web-Based Survey

**DOI:** 10.1155/2022/8919830

**Published:** 2022-10-03

**Authors:** Hiba M. Hussien, Zena Hekmat Altaee, Mohammed Nahidh, Sajid Chaffat Auliawi Al-Mayahi

**Affiliations:** ^1^Department of Orthodontics, College of Dentistry, University of Baghdad, Baghdad, Iraq; ^2^Department of Orthodontics, College of Dentistry, University of Anbar, Anbar, Iraq; ^3^Specialist Orthodontist, Ministry of Health, Baghdad, Iraq

## Abstract

**Objectives:**

This study evaluated the use and preference of different types of functional appliances among a sample of Iraqi orthodontists.

**Materials and Methods:**

About 200 orthodontists were invited to fill out an online Google form questionnaire with multiple-choice questions. The questions were modified from previous England and Malaysian studies. The data were tabulated as frequency tables.

**Results:**

The response rate was 61%. About 91.80% of the participants used functional appliances, with the removable type being the most used. The twin block was thought to be the best compliance one but not the most used functional appliance. About 62% depended on the clinical observations in determining the growth spurt and asked patients to wear the appliance full-time except at meal time. The majority preferred a period of retention of about 4–6 months and frequent visits for adjustments.

**Conclusions:**

Removable functional appliances are the most frequently used among the studied sample.

## 1. Introduction

“Orthodontics is not only the appliance, but it is about which appliances, why, when, and for how long.” The stomatognathic system is a living, viable, and remarkably adaptive system, mainly during its growth and development. Bone is one of the most responsive tissues to environmental stimuli. Despite that, it is the hardest tissue in the human body [1].

Orthodontic functional appliances might be one of two types: removable or fixed, mainly used to improve the position of the retruded lower jaw in relation to the upper one by directing the forces of stretching the muscles of the face, fascia, as well as the periodontium [2].

Variable compliance expectations placed on the patient are the primary management distinction between these two. The removable one necessitates attention to wear instructions, whereas fixed functional appliances necessitate patient assistance in preventing breakages and maintaining an exceptional level of oral hygiene [3].

The late mixed or early permanent dentition phase is optimal for using functional appliances. An earlier indication may be required, such as when preventative measures against psychological trauma are needed [4]. On the other hand, patients with a vertical growth propensity or who are nongrowing are contraindications for this form of orthodontic treatment. Cases with mandibular backward growth rotation, an anterior open bite, and a high mandibular plane angle should be treated cautiously [5].

It is claimed that functional appliances stimulate mandibular growth [6, 7]. Most clinical outcomes are stated to be dentoalveolar alterations, with 70 percent of overjet decrease attained by incisor tipping [8]. Bad oral habits such as bruxism can also be treated with these appliances in addition to some prosthodontic appliances [9].

Recently, the utilization of teledentistry that uses telecommunication technology such as dental videos, images, and electronic records has risen, especially in the era of COVID-19. This modern technology facilitates the explanation of different problems in medicine and dentistry [10–12] and also enables orthodontists to explain the shape, mode of action, and uses of different orthodontic appliances.

Iraq's orthodontics clinics have access to many commercially available functional appliances. The clinician's preference and selection are determined mainly by the education, abilities, experiences, and emphasis acquired throughout orthodontic training.

This survey is performed to explore the variance in the application and management protocols of functional appliances by Iraqi orthodontists, as well as to clarify the clinical preferences among providers.

## 2. Materials and Methods

### 2.1. Study Design and Approval

Approval of the scientific committee in the Department of Orthodontics, the College of Dentistry, University of Baghdad was gained to conduct this cross-sectional web-based survey among Iraqi orthodontists.

### 2.2. Sample

About 200 Iraqi orthodontists registered in the Iraqi orthodontic society were invited to participate in this online study.

### 2.3. Methods

A survey on Google forms was prepared, and it consisted of general questions about the use and preference of functional appliances utilized by Iraqi orthodontists to treat different malocclusions. This questionnaire was modified from Chadwick et al. [13] and Bahar et al. [14] research works, and it included the following questions in addition to the personal data:

“Do you use functional appliance therapy in your practice?”

- Yes
- No

#### 2.3.1. If No

1. “What are the reasons for not offering functional appliance treatment?”

- I do not have adequate experience with the functional appliance
- Poor patient cooperation in most of the cases
- I do not believe that it causes major skeletal changes rather than dental

(2) “Do you refer such a patient to one more orthodontist for that treatment?”

- Yes
- No

#### 2.3.2. If Yes

Orthodontists' preferences of functional appliances in treating different malocclusions are as follows:

1. “Generally, which type of functional appliance do you frequently use?”

- Fixed
- Removable
- Semifixed
- A and B
- All

(2) “What is (are) the preferred fixed functional appliance(s) you used?”
(A) Herbst
(B) Jasper jumper
(C) Sabbagh universal spring (SUS)
(D) The mandibular anterior repositioning appliance (MARA)
(E) Others
(F) I do not use this type

(3) “What is (are) the preferred removable functional appliance(s) you used in managing class II cases?”

- Monobloc
- Bionator
- Twin block
- Inclined anterior bite plane
- Frankel II
- Myobrace

(4) “What is (are) the preferred removable functional appliance(s) you used in managing class III cases?”

- Monobloc
- Frankel III
- Bionator
- Myobrace

(5) “Name of the preferred functional appliance(s) you used in managing bad oral habit cases?”

- Oral screen
- Lip bumper
- Myobrace

Limitations with the functional appliance are as follows:

1. “What are the factors limiting your choice for the removable functional appliance?”

- Availability of a well-qualified technician
- Availability of laboratory support
- Compliance of the patients
- Financial status of the patients
- Age of the patients
- Severity of the case itself

(2) “What are the factors limiting your choice for the fixed functional appliance?”

- Availability of the appliance
- Experience of the orthodontist
- Financial status of the patients
- Age of the patients
- Severity of the case itself

Treatment protocol:

1. “At what age do you typically commence functional appliance treatment for class II cases?”

- As early as detected
- Before the maximum growth spurt
- At the maximum growth spurt

(2) “At what age do you typically start functional appliance treatment for class III cases?”

- As early as detected
- Before the maximum growth spurt
- At the maximum growth spurt

(3) “What is the method of determining the skeletal maturation?”

- Appearance of secondary sexual characteristics such as hair on the face in males and the menstrual cycle in females
- Depending on the lateral cephalometrics for assessing the cervical vertebra maturation (CVM) stages
- Depending on the hand-wrist X-ray for assessing the maturation of the middle phalanx
- Depending on the OPG or periapical X-ray to assess teeth maturation

(4) “Do you think that cases that need potential growth modification are referred to you at an ideal time?”

- Frequently
- Sometimes
- Rarely

(5) “What is your classic wearing regime for the removable functional appliances?”

- Full time including meal times
- Full time NOT including meal times
- Part time

(6) “Do you give a chart or diary to your patient to assess his/her compliance with removable appliance?”

- Yes
- No

(7) “Based on your experience in using functional appliances, what is the best compliance appliance for the patients?”

- Monobloc
- Bionator
- Twin block
- Frankel
- Myobrace
- Fixed type

(8) “Do you give a retention period following active removable functional appliance therapy when the appliance is worn less?”

- Yes
- No

(9) “If so, how long will it last?”

- Two–three months
- Four–six months
- Seven–nine months
- >Nine months

(10) “Do you perform any adjustment to the removable functional appliance during this retention phase?”

- Yes
- No

### 2.4. Statistical Analysis

Responses were collected and analyzed by using the statistical packages for the social sciences (SPSS) program version 25 released in 2017 from IBM SPSS statistics cooperation, USA. The frequency and percentage of responses were tabulated.

## 3. Results

Table 1 shows the demographic distribution of the participants' data. The male-to-female ratio is nearly equal, with a 61% response rate (122 from 200 orthodontists invited). Most of the participants were awarded the Master of Science degree in orthodontics, and more than 70% are working in public hospitals and private clinics.

Upon asking about using the functional appliances in daily practice, 112 (91.80%) responded positively, while only 10 participants did not use the functional appliance due to lack of adequate experience or poor patient cooperation. Of those ten, only 6 refer the patients to other orthodontists (Table 2).

### 3.1. Orthodontists' Preferences of Functional Appliances in Treating Different Malocclusions

Referring to Table 3, about 73.21% prefer to use the removable appliance, two only used the fixed one, and 28(25%) used both appliances.

Of 30 participants who used the fixed appliance, 5 preferred Herbst, 4 preferred SUS, 3 preferred MARA, 3 preferred Herbst and MARA, and 15 used other types.

The most preferred removable appliances for treating class II cases are twin block, myobrace, and monobloc, while for class III, myobrace and Frankel III are the most preferred. In addition, myobrace is preferred as a habit breaker.

### 3.2. Limitations with Functional Appliance

**Figure 1:**
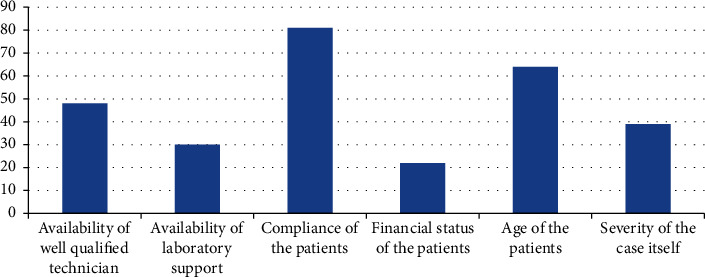
Factors limiting orthodontists' choice for the removable functional appliance.

**Figure 2:**
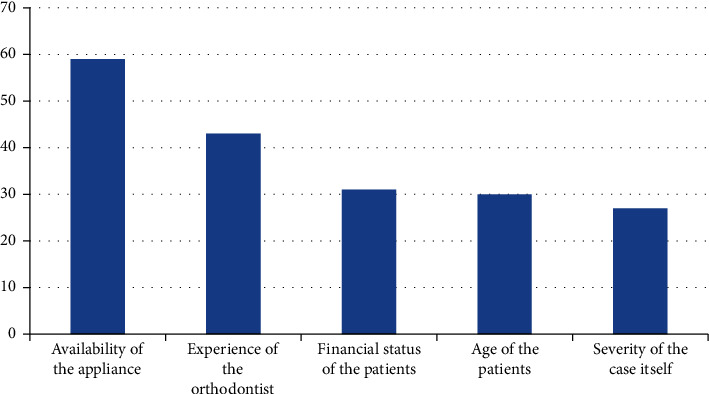
Factors limiting orthodontists' choice for the fixed functional appliance.

Reviewing Figure 1, the most important limiting factors with removable functional appliances are the compliance and the age of the patients at the time of treatment and the availability of laboratory support. Alternatively, the limiting factors with the fixed functional appliance are the availability of the appliance and the experience of the orthodontists (Figure 2).

### 3.3. Treatment Protocol

About 49.11% of the participants treated Class II cases with the functional appliance before the maximum growth spurt, while 32.14% preferred the treatment at the maximum growth spurt (Table 3).

On the other hand, for Class III cases, 69.64% treated their cases as early as detected, while 30.36% postponed the treatment to the period before the maximum growth spurt.

Twin block appliance had the highest rate of patient complaints (29.51%), followed by the fixed type (28.69%), then myobrace (22.13%), and monobloc (16.39%).

Regarding the methods of determining skeletal maturation, 54.46% of the participants depend on the appearance of secondary sexual characteristics such as hair on the face in males and the menstrual cycle in females. In comparison, 17.86% depend on the OPG and lateral cephalometric radiographs to determine the CVM, yet only 9.82% use the hand wrist for that purpose.

Regarding case referral at the ideal time, 65.18% responded that sometimes the cases that need growth modification are referred at the ideal time, against 23.21% that are rarely referred at the ideal time. The applied wearing regimen for the removable functional appliances was 54.55% full time, excluding meal times, while 40% preferred the part-time regimen. To assess patient acquiescence with removable functional appliance therapy, a diary or chart is only applied by 31.82% of the participants.

About 98.18% of the participants used the removable functional appliances as retainers if worn less than required, and the highest retention period extends between 4 and 6 months, and 72.73% made adjustments to these appliances (Table 3).

## 4. Discussion

Functional appliances are orthodontic appliances used to modify the growth and establish the normal function of the perioral muscles. Their effects are primarily dentoalveolar [8]. These appliances could be classified into four categories, namely, the passive tooth-borne like activator and bionator, the active tooth-borne, e.g., twin block, fixed functionals, the tissue-borne like Frankel regulators series, and the combined one as the hybrid appliance [15].

By using these appliances at the appropriate time indicated that patients may reduce the necessity for future orthognathic surgeries. Hence, the assessment of the preference and use of functional appliances among Iraqi orthodontists is the aim of the current study, which is considered the first in Iraq and the Middle East and the third in the world after Chadwick et al. [13] in the U.K. and Bahar et al. [14] in Malaysia.

An invitation to participate in this study was sent to about 200 Iraqi orthodontists. Just 122 orthodontists participated with a 61% response rate. This is lower than Chadwick et al. [13], which was 87.7%, and higher than Bahar et al. [14], which was 39.3%. The gender distribution was nearly equal, with the majority awarded a master's degree in orthodontics. About 70% of the participants worked in private and public clinics (Table 1).

Among Iraqi orthodontists, a high rate (91.8%) used functional appliances to treat different malocclusions, as these appliances are efficient in the early correction of some skeletal problems. This percentage is comparable to that in previous studies [13, 14]. The cause of not using it by 8.2% of participants was either lack of experience or poor patient cooperation; 60% of those would refer the patient to another orthodontist, while the remaining percent preferred other treatment options rather than functional appliances (Table 2).

### 4.1. Orthodontists' Preferences of Functional Appliances in Treating Different Malocclusions

Referring to Table 3, among 112 participants who used the functional appliances, 73.21% preferred using removable functional appliances, which can be explained by the low cost of this type, good patient compliance, and more control of oral hygiene when compared to the fixed type, while about 25% preferred both removable and various types of fixed appliances, and only two participants used the fixed type utterly. These results are similar to the results of the survey among orthodontists in Malaysia [14].

Functional orthodontic treatment influences mandibular position and function by advancing the mandible relative to its typical resting position, altering muscular conditions, and reducing Class II disparity [7, 16]. Concerning the current survey, most of the participants preferred the use of various types of appliances; specifying the appliance type, monobloc and myobrace are the most popular functional appliances among Iraqi orthodontists before or at the maximum growth spurt, followed by the twin block appliance, which disagreed with other studies from different communities that clarify the preference of twin block appliance due to its high efficiency by keeping the jaw in a forward posture to induce favorable growth of the condyle and hence correction of Class II malocclusion [13, 14]; this difference might be simply due to orthodontists preference or to poor available laboratory techniques in Iraq.

For managing Class III malocclusion, facial growth modification is considered an effective method of managing jaw inconsistencies in growing children with dentofacial orthopedic appliances, including the face mask to protract the maxilla and the Frankel functional regulator III appliance [17]; moreover, a mixed anchored palatal expander can give beneficial effects in treating class III skeletal problem [18]. As shown by the results of this study, myobrace and Frankel III are the first and second treatment options by most orthodontists, followed by monobloc and bionator, as early as detected and before the maximum growth spurt prior to the closure of the circum-maxillary sutures, to get the full benefit of growth modification and to decrease the chances of future surgical intervention.

Regarding bad oral habit management, the answers to questions showed that the highest preferred appliance is the myobrace, which could be due to its modernity, well-observed results, acceptable patient compliance, in addition to no need for laboratory work of this ready-made appliance, and the other preferred appliance is lip bumper. At the same time, the least preferable one is the oral screen, as it is the most annoying appliance for patients.

### 4.2. Limitations with Functional Appliance

Reviewing Figure 1, the main limitations with removable functional appliances are the compliance and the age of the patients at the time of treatment. Moreover, the availability of laboratory support, especially for Frankel appliances and twin blocks, is also a limiting factor. On the other hand, the limiting factors with the fixed functional appliance are the availability of the appliance and the experience of the orthodontists (Figure 2). So, training programs for lab technicians should be scheduled to take updated information regarding the fabrication of twin blocks and different types of Frankel appliances.

### 4.3. Treatment Protocol

Any orthodontic device is more effective if patients can adapt to it immediately and consistently find it easy to use. If an appliance does not allow for quick acclimation and convenience of usage, patients are unlikely to be compliant, resulting in ineffective orthodontic movements and increased treatment time; assessing appliance acceptance is crucial for assuring compliance and, ultimately, treatment efficacy [17]. According to the participants of this study, the twin block appliance is the best when regarding patient compliance, followed by the fixed functional appliance, which agrees with other surveys in the UK [13] and Malaysia [14]; the other two appliances with relatively high patient compliance are the myobrace and monobloc, and on the other hand, bionator and Frankel appliances are the least tolerated by the patients.

Estimation of skeletal maturation is an essential requirement of a functional appliance treatment plan; the participants of the current survey relied primarily on clinical appearance as it requires the least cost and effort, followed by radiological methods, either lateral cephalometric radiography or orthopantomography, while only 11 participants out of 112 referred their patients for hand-wrist radiography to achieve this purpose, and despite its efficiency in determining skeletal maturation, this low number could be due to increased awareness of the hazards of X-ray, so getting maximum benefits of already available radiographs of the cases without the need for further radiation exposure.

Only a small percentage of orthodontists (11.61%) stated that growth modification cases are frequently referred for treatment at an ideal time, which agreed with the results of the Malaysian survey [14]. In comparison, the majority of participants (65.18%) claimed that this type of case is sometimes referred to as an ideal time, and the remaining (23.21%) percent rarely received these cases at the ideal time; this requires a definite increase in public awareness about the benefit of early intervention and monitoring growth modification cases, including TV programs and social media activities [11, 12].

Considering the functional appliance wear regime, the higher percentage of participants preferred a full-time regime not including meal times in order to ensure that the appliance will not break from hard food; on the other hand, the remaining part preferred a part-time regime, while the least percent preferred full-time regime including meal times. The results of the Malaysian survey [14] confirmed that a full-time wear regime is most dependable.

Despite the worries of orthodontists about wearing the appliance by the patient according to the described regime, only one-third of participants set a chart to evaluate patient compliance with removable functional appliance therapy. At the same time, the majority (68%) considered this tool inapplicable, indicating the need to improve patient education to enhance cooperation and get better results.

Nearly all participants are aware of the relapse problem who followed the treatment by using removable appliances, so they instruct their patients to wear the appliance as a retainer for a relatively acceptable period, mostly 4–6 months, which is different from the retention period applied by Malaysian orthodontists, who preferred 2–3 months of appliance wear after active treatment.

Orthodontic therapies are frequently lengthy, mainly when undesirable side effects arise and the practitioner does not detect them for several months [19]. In order to save time and avoid or, at least, limit the effects of unanticipated complications, it is essential to monitor the process of treatment strictly, regardless of the employed technique [20]; this is also applicable when using removable functional appliances, so the continuous adjustment of removable functional appliances is an important step to ensure its retention inside the patient mouth, with continuous monitoring of the treatment effect; fortunately about 72% of participants are aware of the importance of this step.

One of the study limitations that must be taken with vigilance is that the responses from the participants might not reproduce the attitudes and practices of all orthodontists in Iraq; moreover, the lab staff should be trained well to fabricate twin blocks because of the wide popularity of this appliance worldwide in addition to its advantages over other appliances.

## 5. Conclusions

The removable functional appliances are the most used ones. More theoretical and clinical training about fixed functional appliances is required for Iraqi orthodontists. Experienced technicians in twin blocks and advanced lab technologies are also needed. Moreover, continuous education programs regarding functional appliances should be arranged to review this topic and its update.

## Figures and Tables

**Table 1:** Demographic data of the participants.

Parameters		*N*	%
Gender	Male	63	51.64
	Female	59	48.36
	Total	122	100

Qualification	Certificate	8	6.56
	M.Sc.	96	78.69
	Ph.D.	18	14.75
	Total	122	100

Place of work	Public hospital	11	9.02
	Private	25	20.49
	Both	86	70.49
	Total	122	100

**Table 2:** Response of participants' usage of the functional appliances.

Questions	Answers	*N*	%
Do you use functional appliance therapy in your practice?	Yes	112	91.80
	No	10	8.20
	Total	122	100

What are the reasons for not offering functional appliance treatment?	Lack of experience	5	50
	Poor patient cooperation	5	50
	Total	10	100

Do you refer such a patient to one more orthodontist for that treatment?	Yes	6	60
	No	4	40
	Total	10	100

**Table 3:** Appliance preference, diagnosis, wearing, and retention regimen.

Questions	Answers	*N*	%
Generally, which type of functional appliance do you frequently use?	Removable	82	73.21
	Fixed	2	1.79
	Semi-fixed	0	0
	Both	28	25
	Total	112	100

What is (are) the preferred fixed functional appliance(s) you used?	Herbst	5	16.67
	MARA	3	10
	SUS	4	13.33
	Herbst and MARA	3	10
	Others	15	50
	Total	30	100

What is/are the preferred removable functional appliance(s) you used in managing Class II cases?	Monobloc	12	10.91
	Bionator	2	1.82
	Twin block	10	9.09
	Inclined anterior bite plane	3	2.73
	Frankel II	1	0.
	Myobrace	11	10
	More than one appliance	71	64.5
	Total	110	100

What is/are the preferred removable functional appliance(s) you used in managing Class III cases?	Monobloc	14	12.73
	Frankel III	34	30.91
	Bionator	8	7.72
	Myobrace	43	39.09
	Bionator and myobrace	2	1.82
	Frankel III and myobrace	9	8.18
	Total	110	100

Name of the preferred functional appliance(s) you used in managing bad oral habit cases.	Oral screen	21	18.75
	Lip bumper	28	25
	Myobrace	47	41.96
	All	16	14.29
	Total	112	100

At what age do you typically commence functional appliance treatment for Class II cases?	As early as detected	21	18.75
	Before the maximum growth spurt	55	49.11
	At the maximum growth spurt	36	32.14
	Total	112	100

At what age do you typically start functional appliance treatment for Class III cases?	As early as detected	78	69.64
	Before the maximum growth spurt	34	30.36
	At the maximum growth spurt	0	0
	Total	112	100

What is the method of determining skeletal maturation you usually used?	Clinical	61	54.46
	Hand-wrist	11	9.82
	Lateral ceph.	20	17.86
	OPG	20	17.86
	Total	112	100

Do you think that cases that need potential growth modification are referred to you at an ideal time?	Frequently	13	11.61
	Sometimes	73	65.18
	Rarely	26	23.21
	Total	112	100

What is your classic wearing regime for the removable functional appliances?	Full-time including meal times	6	5.45
	Full-time not including meal times	60	54.55
	Part-time	44	40
	Total	110	100

Do you give a chart or diary to your patient to assess his/her compliance with the removable appliance?	Yes	35	31.82
	No	75	68.18
	Total	110	100

Based on your experience in using functional appliances, what is the best compliance appliance for the patients?	Monobloc	20	16.39
	Bionator	2	1.64
	Twin block	36	29.51
	Frankel	2	1.64
	Myobrace	27	22.13
	Fixed type	35	28.69
	Total	122	100

Do you give a retention period following active removable functional appliance therapy when the appliance is worn less?	Yes	108	98.18
	No	2	1.82
	Total	110	100

If so, how long will it last?	2–3 months	17	15.74
	4–6 months	50	46.30
	7–9 months	7	6.48
	>9 months	34	31.48
	Total	108	100

Do you perform any adjustments to the removable functional appliance during this retention phase?	Yes	80	72.73
	No	30	27.27
	Total	110	100

## Data Availability

The data used to support the findings of this study are available from the corresponding author upon request.
